# Characteristics of microbial communities and intestinal pathogenic bacteria for migrated *Larus ridibundus* in southwest China

**DOI:** 10.1002/mbo3.693

**Published:** 2018-07-05

**Authors:** Feng Liao, Wenpeng Gu, Duo Li, Junrong Liang, Xiaoqing Fu, Wen Xu, Ran Duan, Xin Wang, Huaiqi Jing, Jiejie Dai

**Affiliations:** ^1^ Department of Respiratory Medicine The First People's Hospital of Yunnan Province Kunming Yunnan China; ^2^ The Affiliated Hospital of Kunming University of Science and Technology Kunming Yunnan China; ^3^ Center of Tree Shrew Germplasm Resources Institute of Medical Biology The Chinese Academy of Medical Science and Peking Union Medical College Yunnan Key Laboratory of Vaccine Research and Development on Severe Infectious Diseases Yunnan Innovation Team of Standardization and Application Research in Tree Shrew Kunming China; ^4^ Department of Acute Infectious Diseases Control and Prevention Yunnan Provincial Centre for Disease Control and Prevention Kunming China; ^5^ National Institute for Communicable Disease Control and Prevention Chinese Center for Disease Control and Prevention State Key Laboratory for Infectious Disease Prevention and Control Beijing China

**Keywords:** gut microbiota, intestinal pathogenic bacteria, *Larus ridibundus*, southwest China

## Abstract

*Larus ridibundus*, a migratory wild bird, has become one of the most popular gull species in southwest China in recent years. There has been no information on the gut microbiota and intestinal pathogenic bacteria configuration in wild *L. ridibundus*, even though the public are in close contact with this bird. In this study, 16S rRNA amplicon‐sequencing methods were used to describe the microbial community structure and intestinal pathogenic bacteria were isolated to identify their characteristics. The taxonomic results revealed that Firmicutes (86%), Proteobacteria (10%), and Tenericutes (3%) were the three most abundant phyla in the gut of *L. ridibundus*. *Enterococcaceae*,* Enterobacteriaceae*, and *Mycoplasmataceae* were the most predominant families, respectively. The number of operational taxonomic units (OTUs), the richness estimates and diversity indices of microbiota, was statistically different (*p* < 0.05). However, beta diversity showed that no statistical significance (*p* > 0.05) between all the fecal samples. The most frequently isolated intestinal pathogenic bacteria from *L. ridibundus* were enteropathogenic *Escherichia coli* (32%) and *Salmonella* (21%). Pulsed‐field gel electrophoresis (PFGE) results of *Salmonella* species revealed a high degree of similarity between isolates, which was not observed for other species. None of the potentially pathogenic isolates were identical to human‐isolated counterparts suggesting that there was little cross‐infection between humans and gulls, despite close proximity. In brief, this study provided a baseline for future *L. ridibundus* microbiology analysis, and made an understanding of the intestinal bacterial community structure and diversity.

## 1 INTRODUCTION

*Larus ridibundus* is a migratory bird distributed in Eurasia and on the East Coast of North America. Its body and coat color is similar to pigeons, showing red beak and feet, and the feathers of most of the body are white (Rajaei, Esmaili Sari, Bahramifar, Ghasempouri, & Savabieasfahani, 2010; Ushine, Sato, Kato, & Hayama, 2017). Xinjiang, Inner Mongolia, Heilongjiang and other places in China are their breeding grounds. Every winter flocks of *L. ridibundus* migrate from Siberia to Southern China to overwinter; and their overwintering places are widely distributed from Northeast to Southeast China. Since the 1980s, every year from November to March, tens of thousands *L. ridibundus* fly to Kunming (southwest China) to overwinter. Each year, many visitors come to watch and feed them on city lakes or rivers; and thus the public are in close contact with this wild bird. Therefore, we speculated and studied the possibility there may be intestinal pathogenic bacteria from *L. ridibundus* that may endanger public health (Broman et al., 2002; Sixl et al., 1997).

Metagenomic investigations can be used to estimate the distributions of microorganisms, the taxonomic diversity, and functional gene contents (Cornejo‐Granados et al., 2017). Studies in the past on microbiota were performed by using culture‐based methods, and research result in the field was limited. However, recent availabilities in next‐generation sequencing (NGS) methods have allowed a more thorough analysis of the complex and diverse of gut microbial communities (Kwong, McCallum, Sintchenko, & Howden, 2015). Therefore, a large number of studies on the gut metagenomics, using NGS system have been reported since then, which provided a broad and deep understanding of the microbial community. Previous studies showed that gut microbial mutualisms, commensalisms, and pathogen interactions have been considered to be an important factor for human health and animals (Backhed, Ley, Sonnenburg, Peterson, & Gordon, 2005). Several studies on gut metagenomic analysis have been conducted on some domestic bird species (Waite & Taylor, 2014); and some referred to wild birds (Dewar, Arnould, Krause, Dann, & Smith, 2014; Dewar et al., 2013). So far, there has been no information on the gut microbiota and intestinal pathogenic bacteria configuration in migrated wild *L. ridibundus*. In this study, microbial communities of recently migrated *L. ridibundus* were analyzed by 16S rRNA amplicon sequencing investigation, and intestinal pathogenic bacteria were isolated to identify their characteristics.

## 2 MATERIALS AND METHODS

### 2.1 Sample collection of and DNA extraction

Five hundred fecal samples from *L. ridibundus* were collected in Kunming (103°40′ E, 26°22′ N), southwest China. The collection was divided into two periods, November and December 2017. The samples were collected around 5–10 m far from each other along the Daguan River in the center of Kunming city. Each sample was handled in two ways; one was by isolation using methods for common intestinal pathogenic bacteria; the other was randomly selecting 86 samples to extract total DNA for 16S rRNA amplicon sequencing analysis. The whole genomic DNA was extracted by using the fecal samples’ DNA extraction kit (Tiangen, Beijing) following the manufacturer's instructions. In this kit, the genomic DNA of fecal samples was extracted by centrifugal adsorption column with specific binding to total DNA. A unique inhibitor adsorption tablet (InhibitEX), combined with a specially developed buffer system was also used for extraction. The entire microorganism's DNA could be extracted efficiently from fecal samples by using this kit. All extracted DNA samples were stored at −20°C for microbial community analysis.

### 2.2 PCR, amplification, Miseq library construction, and sequencing

The variable region V3–V4 of the 16S rRNA gene was used for the library construction of the bacterial community by Illumina Miseq sequencing. Standard amplicon primers were used (Klindworth et al., 2013), and Illumina adapter overhang nucleotide sequences were added to the gene‐specific sequences according to the protocol of library preparation guideline. PCR amplifications were performed using KAPA HotStart PCR kits (Kapa, Biosystems). Each PCR reaction included 0.2 M Trehalose, 5 μl buffer, 0.75 μl dNTP mixtures, 0.3 μM of the primers, 0.5 U KAPA polymerase, 25 ng extracted DNA, and PCR water totally added up to 25 μl. The amplification conditions were as follows: 95°C for 3 min, followed by 25 cycles of 95°C for 30 s, 55°C for 30 s, and 72°C for 30 s, and final extension at 72°C for 5 min (Bobrova, Kristoffersen, Oulas, & Ivanytsia, 2016). The PCR products were purified with AMPure XP magnetic beads (Beckman, Coulter) and quantified using the Qubit fluorometer (Invitrogen, Life Technologies). A secondary amplifications to attach the Illumina Nextera barcodes were then performed, using the i5 forward primer and i7 reverse primer following the manufacturer's instructions (Kim, An, Kim, Lee, & Cho, 2017). The nontarget fragments were removed, using AMPure XP magnetic beads as mentioned above (Beckman, Coulter). The amplicons were normalized, pooled, and sequencing was conducted in our laboratory (Kunming, China) using an Illumina MiSeq sequencing system (Illumina, San Diego, USA).

### 2.3 Bioinformatics and statistical analysis

The raw reads were firstly trimmed for a quality check and filtering of low quality (<Q25) reads. The paired‐end sequences (310 bp) were then merged by using CLC Genomics Workbench 9.5.2 (QIAGEN, Denmark) (Ashburner et al., 2000; Mevada, Patel, Pandya, Joshi, & Patel, 2017; Misner et al., 2013). After read trimming, the reads were retained and analyzed, using CLC Microbial Genomics Module (version 1.6.1) (Lindgreen, Adair, & Gardner, 2016) of Genomics Workbench. CLC Microbial Genomics Module and CLC Genomics Workbench performed operational taxonomic units (OTU) clustering and estimated alpha and beta diversities in microbial samples.

All the sequences were clustered into OTUs, based on 97% sequence similarity against the Greengenes reference sequence collection (version 13.5) (Cornejo‐Granados et al., 2017). Taxonomy summaries with relative abundance data were generated. The representative sequences were aligned to the Greengenes reference alignment, and the alignment was filtered, then a phylogenic tree was constructed with the UPGMA for tree building. Prior to statistical analysis, the sequencing depths between samples were normalized. Alpha and beta diversity metrics from the final OTUs table were obtained. The alpha diversity analysis, including rarefaction curve and diversity indices, was carried out. The beta diversity was analyzed based on Fast UniFrac (Hamady, Lozupone, & Knight, 2010). Differences in the alpha and beta diversity were investigated, including the number of OTUs, richness, and diversity. Furthermore, differences in taxonomic composition were analyzed from the phylum to the family level. Statistical analysis was performed using a Kruskal–Wallis *H* test in SPSS statistics package, version 16.0 (SPSS IBM, New York, NY, USA), and PERMANOVA analyzed two groups of sampling time (November and December) for multiple testing correction by CLC Microbial Genomics Module (version 1.6.1). *p* value of <0.05 was recognized as statistical significance. Sequence data were deposited on the NCBI database by the SRA accession: SRP131711.

### 2.4 Isolations of the intestinal pathogenic bacteria

The intestinal pathogenic bacterial spectrum included diarrheagenic *E. coli*, nontyphoidal *Salmonella*,* Shigella* spp., *Vibrio* spp., *Aeromonas* spp., and *Plesiomonas* spp. (Yu et al., 2015). All of the samples were first inoculated on Mac Conkey Agar and Xylose Lysine Desoxycholate (XLD) agar (Luqiao, Beijing) and incubated at 37°C for 24 hr. Another portion was inoculated into Selenite Brilliant Green Broth (SBG) and Buffered Peptone Water (BPW) (Luqiao, Beijing) and incubated at 37°C for 24 hr; and then inoculated on Salmonella Shigella agar (SS) and Thiosulfate citrate bile salts sucrose agar (TCBS) (Luqiao, Beijing), and incubated at 37°C for 24 hr. Enrichment was performed using phosphate‐buffered saline with sorbitol and bile salts (PSB) incubating at 4°C for 21 days, then inoculated onto Yersinia‐selective agar (cefsulo‐din‐irgasan‐novobiocin [CIN] agar; Difco) for isolation of *Yersinia*. All suspected entero‐pathogen colonies were picked and identified using Vitek Compact 2 biochemical identification instrument (bioMérieux). Further, all of the isolated *E*. *coli* were identified, using the multiplex PCR diagnostic kit (ABTechnology, Beijing) for the diarrheogenic *E*. *coli*.

### 2.5 Pulsed‐field gel electrophoresis

PFGE was used to analyze the important isolated strains (diarrheogenic *E*. *coli*,* Salmonella* and *Yersinia enterocolitica*) from *L. ridibundus*. The procedures were performed according to the protocols of each bacteria published by PulseNet (Curran et al., 2017; Ferrari, Panzenhagen, & Conte‐Junior, 2017; Liang et al., 2014). The plugs digested with different enzymes, and electrophoresed with a recommendatory pulse times and hours at 20 V. For data analysis, *.tiff images of the gels were imported into the database of PFGE patterns. Clustering of the band patterns was performed, using BioNumerics software (version 6.6) with the unweighted‐pair group method of average linkages (UPGMA) and the Dice coefficient at a 1.5% tolerance. The diarrhea patients’ PFGE patterns for different surveillance pathogenic bacteria from our previous database were used for comparison purpose.

## 3 RESULTS

### 3.1 Taxonomic results of the *L. ridibundus* gut microbiota

**Figure 1:**
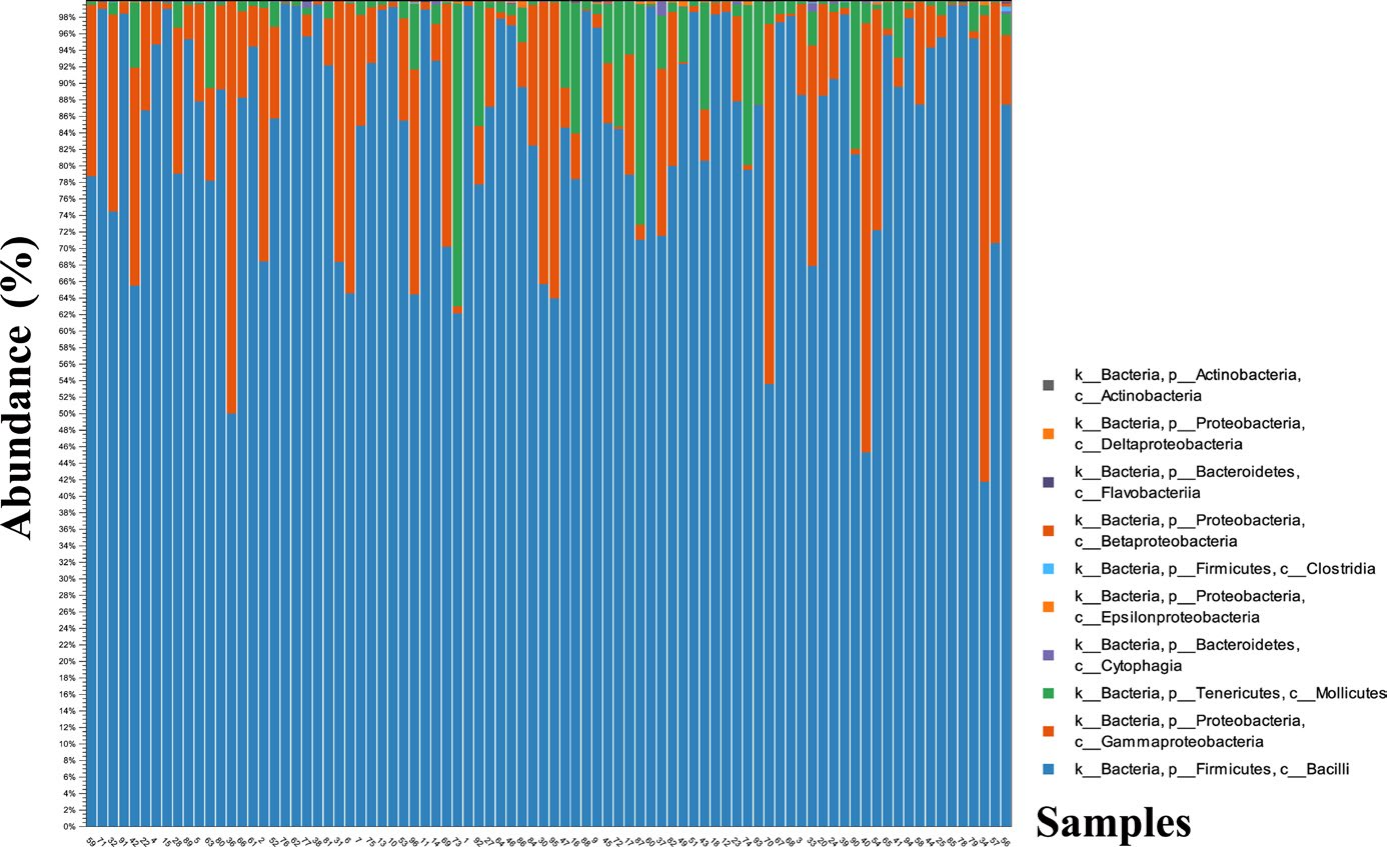
The relative abundance of the *Larus ridibundus* gut microbial communities for each sample

In total, 18,945,847 reads were obtained from all the fecal samples. After quality trimming, merging, primer trimming, and length trimming, 15,143,601 valid reads were obtained from 86 samples. However, 15,013,198 reads were found in OTUs, and total 1,330 predicted OTUs were obtained, included 913 based on database, and 417 de novo. At the phylum level, three major different bacterial phyla in 86 samples were found and constituted Firmicutes (86%), Proteobacteria (10%) and Tenericutes (3%). At the class level, *Bacilli*,* Gammaproteobacteria* and *Mollicutes* comprised most of the microbial communities. At the family level, *Enterococcaceae* (97%), *Enterobacteriaceae* (92%) and *Mycoplasmataceae* (95%) were the major microbiota in each order. The relative abundance of microbial communities for each sample was shown in Figure 1. The constitutions of microbiota for each sample were similar with total distribution, and the most comprised phyla for each sample were Firmicutes, Proteobacteria, and Tenericutes, especially for Firmicutes (Figure 1).

### 3.2 Phylogenic analysis of OTUs

**Figure 2:**
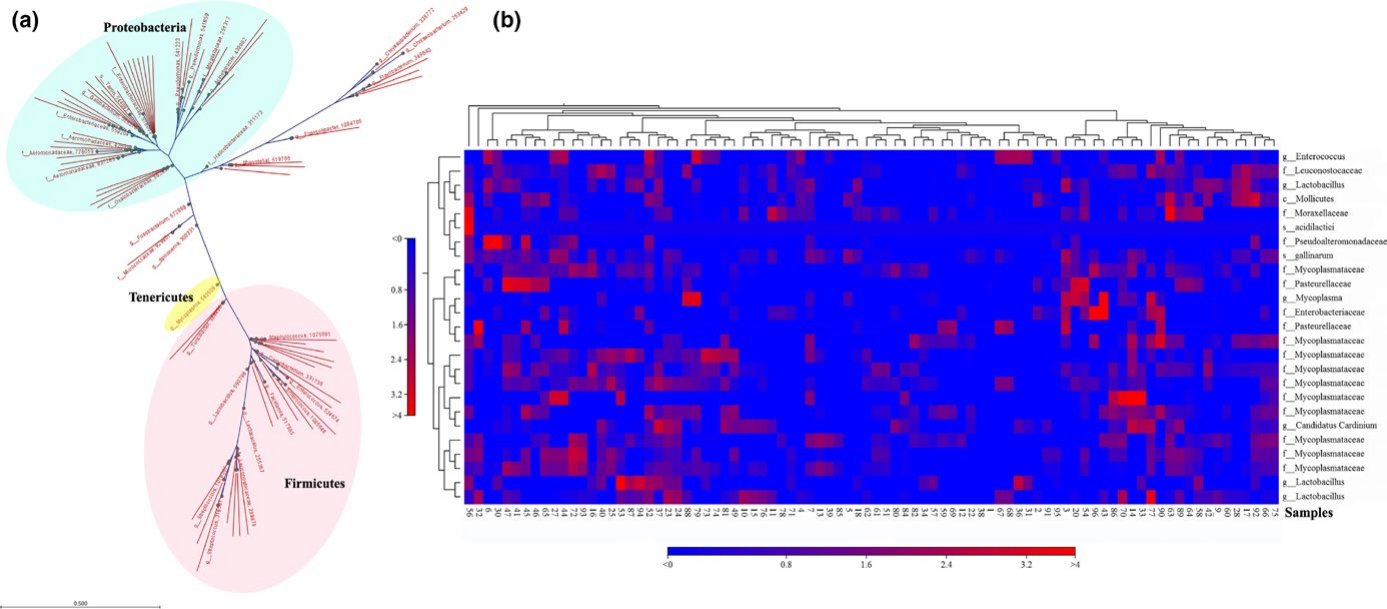
Dendrograms of phylogenic analysis and heat map of the relative abundance for representative family or genus in OTUs. (a) Phylogenic analysis of OTUs. (b) The heat map of the relative abundance for representative family or genus in OTUs

The representative family or genus of OTUs was showed in Figure 2a. Three major cluster groups were found in OTUs alignment, represented three important phyla, pink area belonged to Firmicutes, green area referred to Proteobacteria, and yellow area was Tenericutes. *Enterococcus*,* Lactobacillus*,* Staphylococcus*, and *Streptococcus*, etc. were predominant genus in Firmicutes phylum; *Enterobacteriaceae*,* Aeromonadaceae*, and *Moraxellaceae* were predominant family in Proteobacteria; *Mycoplasma* was the major genus belonged to Tenericutes. The top 25 abundant families or genus levels were shown in heat map (Figure 2B) frequency distribution indicated high or low abundance distributions of microbial communities for each sample.

### 3.3 Diversity estimation

For the alpha diversity analysis, the numbers of OTUs, Chao1, Shannon entropy and Simpson's index of each sample were calculated, then statistical analysis performed using the SPSS software package with Kruskal–Wallis *H* test, revealing that diversity estimates microbiota were significantly different between all the samples (*p* < 0.05) (Table 1). The minimum value of numbers of OTUs was 9.43, and max value was 177.43, showing a large difference.

**Table 1:** The alpha diversity estimation of sequencing library from Miseq sequencing analysis

Indexes	Values (mean ± *SD*)	Min	Max	Kruskal–Wallis	*p* Values
Numbers of OTUs	57.879 ± 24.972	9.430	177.430	1,066	0.000
Chao1	85.247 ± 24.208	31.258	187.012	1,145	0.000
Shannon entropy	0.871 ± 0.529	0.070	2.310	1,624	0.000
Simpson's index	0.665 ± 0.455	0.012	1.953	260.36	0.001

**Figure 3:**
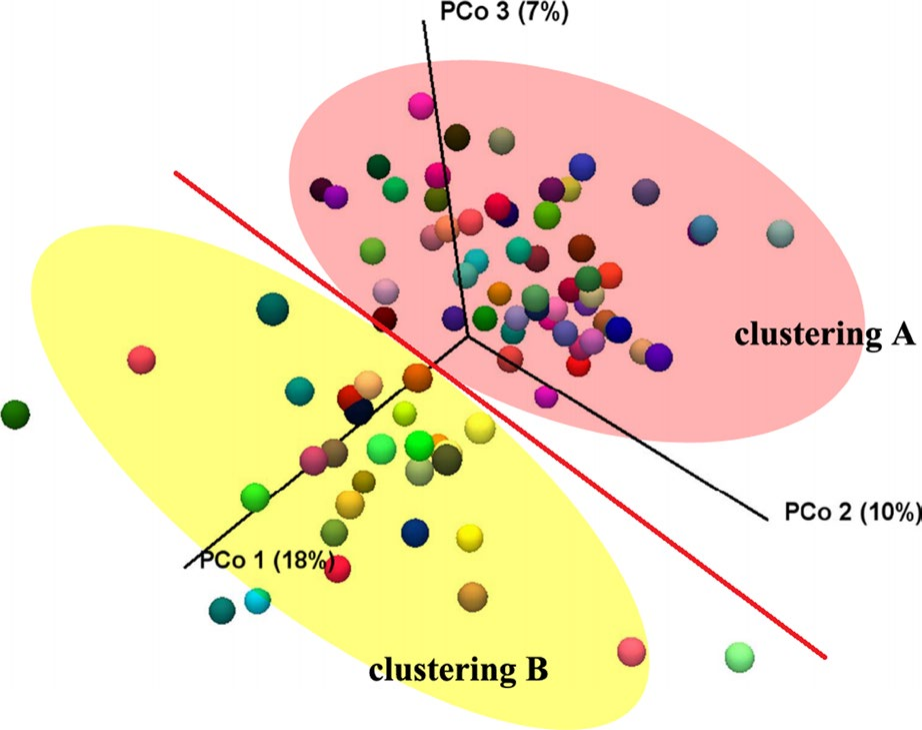
Beta diversity analysis. Unweighted principal coordinate analysis (PCoA) of UniFrac distances. Each color of ball represented each sample in this study. Most samples in pink area of clustering A were collected in December, and yellow area of clustering B in November

PCoA plot according to the Fast UniFrac distance metric was used to investigate the composition of gut microbiota between all the samples. There appeared to be a division across the primary axis for samples distributed in plot, as Figure 3 shown. Most of samples in clustering A were collected in December, while November for clustering B. Therefore, the time of sampling was considered as potential cause for clustering phenomenon. However, PERMANOVA statistical analysis results showed no significance of the information about the distribution, no matter for clustering A (*F* = 1.012, *p* = 0.450), or clustering B (*F* = 1.197, *p* = 0.333).

### 3.4 Pathogenic intestinal bacteria detection results

**Figure 4:**
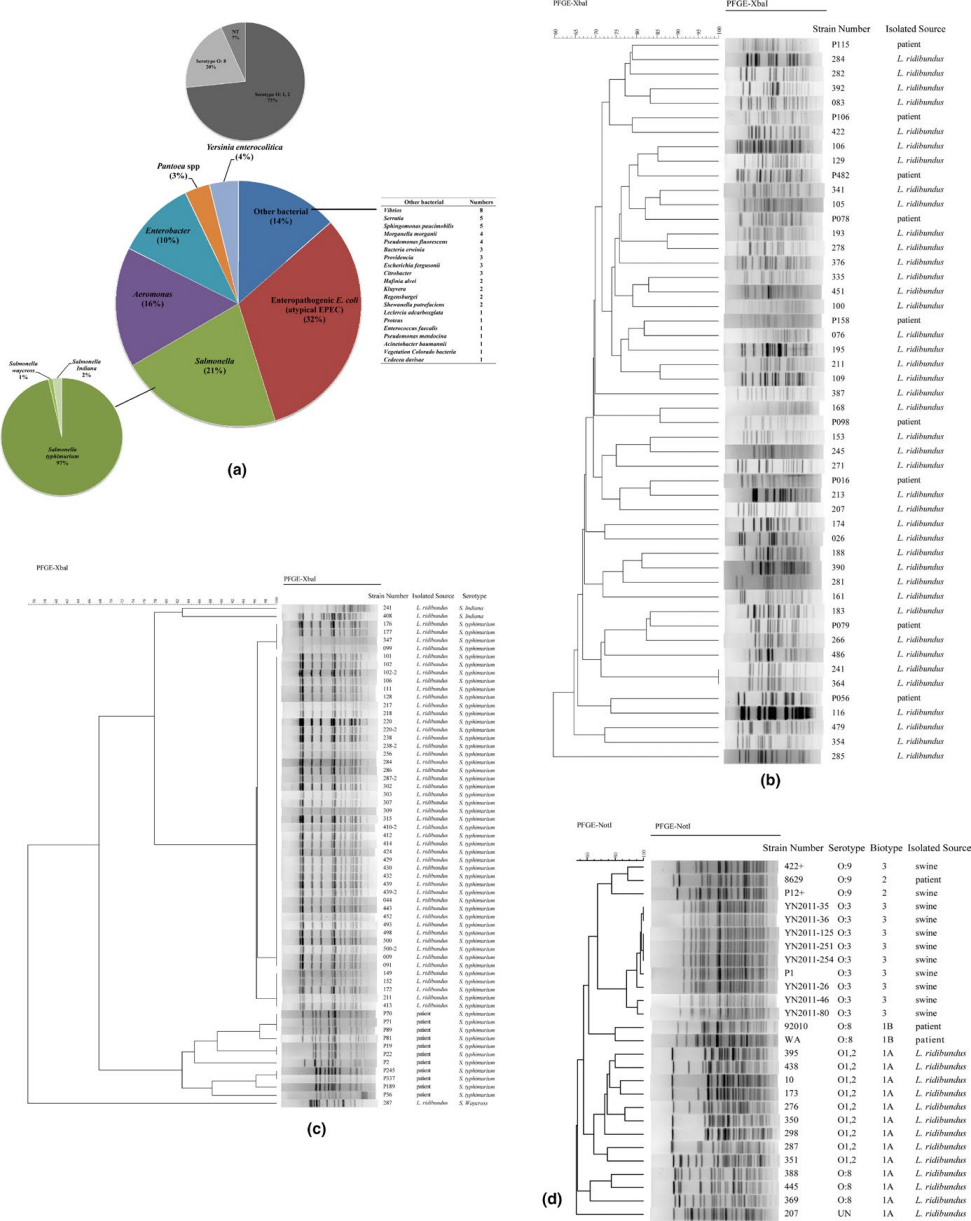
The intestinal bacteria isolation and PFGE clustering results for important pathogenic isolates in this study. (a) The composition of intestinal bacteria isolation. (b) Dendrogram for PFGE patterns of enteropathogenic *Escherichia coli*. (c) Dendrogram for PFGE patterns of *Salmonella*. (d) Dendrogram for PFGE patterns of *Yersinia enterocolitica*

One hundred twenty‐four enteropathogenic *E. coli* (EPEC), 84 *Salmonella*, 62 *Aeromonas*, 41 *Enterobacter*, 15 *Y. enterocolitica*, 13 *Pantoea*, and 38 other bacteria were isolated from the 500 fecal samples. However, EPEC*, Salmonella*, and *Aeromonas* accounted for 69% of all isolated strains (Figure 4a). Interestingly, all isolated EPEC strains were defined as atypical; these strains did not possessed the EAF plasmid (*bfpB* and *escV* gene negative), where the *eae* gene was positive using multiplex PCR. All the *Salmonella* isolated from *L. ridibundus* was nontyphoid *Salmonella* strains, among them, 81 (96.43%) were *Salmonella typhimurium*, one was *Salmonella Waycross*, and two were *Salmonella Indiana*. All of the isolated *Y. enterocolitica* were biotype 1A, and the virulence genes for *ail*,* ystA*,* ystB*,* virF*, and *yadA* were absent. Eleven strains were serotype O: 1, 2; three strains were serotype O: 8; and one strain could not be typed.

### 3.5 PFGE

PFGE results of all the EPEC displayed great heterogeneity, almost no identical PFGE patterns were found for 124 strains. We selected some patients suffering from diarrhea infected by EPEC from our previous study (Zhang, Yang, et al., 2016; Zhang, Zhou, et al., 2016) for comparison purpose, and no similar patterns were found between this wild bird and patients, as Figure 4b shown. For *Salmonella*, 81 *S. typhimurium* were divided into three patterns groups, and the similarity of three patterns groups was greater than 95%. Here, 66 strains showed the same PFGE pattern in the study. Compared with the PFGE patterns with diarrhea patients in our database, we found that *S. typhimurium* isolated from *L. ridibundus* were different from patients, separated into two clustering groups (Figure 4c). The PFGE results showed the 15 *Y. enterocolitica* were in 13 PFGE patterns (Figure 4d). Only two strains had the identical PFGE patterns showing the bird strains had a high diversity. The serotype O: 1, 2, serotype O: 8 and un‐determined strains were separated into different groups based on PFGE patterns. We compared the isolated *Y. enterocolitica* in this study to pathogenic reference strains; the data show the pathogenic and nonpathogenic isolates were divided into two major groups, and the highly pathogenic bio‐serotype 1B/O: 8 and weakly pathogenic O: 3 or O: 9 strains were clustered into one major group.

## 4 DISCUSSION

Gulls are comprised several species, such as *Larus domesticus*,* Larus atricilla*,* Larus audouinii*,* Larus californicus*, and *Larus ridibundus*. Most of the species lived around seashores or rivers, and therefore their feces could be considered as the major source of contamination in coastal and lake waters. Since their migratory character and feeding behavior (Berg & Anderson, 1972), public maybe concerned on the possibility of waterfowl fecal contamination in environmental waters for the potential spread of microbial pathogens to humans, domesticated animals and human food sources. However, our actual data in this study did not strongly support this hypothesis. Previous study (Lu et al., 2011) showed that gull fecal pollution was widespread in urban coastal and riverine areas in North America and that it significantly contributed to fecal indicator bacterial loads. However, the risks associated with exposure to recreational waters impacted by fresh gull, chicken, or pig faeces appear substantially lower than waters impacted by human sources (Soller, Schoen, Bartrand, Ravenscroft, & Ashbolt, 2010). As one of species of gulls, every winter, thousands of *L. ridibundus* flied to warm Kunming from remote Siberia for overwintering where the primary habitats were the lakes or rivers in the city. These areas were centralized areas for tourists and citizens where people could contact *L. ridibundus*. In our study, none of the potentially pathogenic isolates were identical to human isolates counterparts suggested that there was little cross‐infection between human and gulls regardless of the potential close encounters. It was further suggested that the public health risks of gull fecal pollution (at least this species) were less than human and other animal sources.

Several studies showed the gut microbiota of waterfowl, using culture‐based methods and mainly concerned on some pathogens (Craven et al., 2000; Gaukler et al., 2009). Because culture‐based studies only provide a limited finding of natural microbial communities, culture‐independent molecular methods can be used to describe the composition of waterfowl gut microbiome. A study referred to 60 mammalian species showed that microbial communities at higher taxonomic levels were very similar between birds and mammals; and most researches showed two phyla, Firmicutes and Bacteroidetes, as dominant out of 75 known microbial phyla (Ley, Lozupone, Hamady, Knight, & Gordon, 2008). This analysis result was very interesting, since it was believed that the common ancestor of amniotes (reptiles, birds, and mammals) possessed a microbial community mostly comprising Firmicutes and Bacteroidetes (Costello, Gordon, Secor, & Knight, 2010). Another study (Lu, Santo Domingo, Lamendella, Edge, & Hill, 2008) demonstrated that the gull–gut bacterial community was mostly composed of populations closely related to *Bacilli* (37%), *Clostridia* (17%), *Gammaproteobacteria* (11%), and Bacteriodetes (1%) in North America. Their study contained seven species of gulls, but did not involve the *L. ridibundus*. Similar results could be found in our study, the most comprised microbial community was Firmicutes for migrated *L. ridibundus*; however, Bacteroidetes was not the major composition. Actually, in their study, Firmicutes accounted for 54.6% of the microbial community, Proteobacteria for 23%, and Tenericutes was 8.9%. The composition of Firmicutes phyla was lower than ours, and only 85 OTUs were obtained from 282 gull fecal samples. These differences might be due to gull species, living habits, or the environmental factors, while the most important one was different methods to generate the 16S libraries. A clone library method was used in Lu et al. study, and they used different primer compared with ours. In our study, Proteobacteria and Tenericutes phylum were the important communities for this wild bird. A meta‐analysis (Waite & Taylor, 2014) of the avian gut metagenome indicated that consistent with the microbiota of vertebrates in general, the avian gut microbiota was found to harbor mostly OTUs belonging to Bacteroidetes, Firmicutes, and Proteobacteria. The phylum Firmicutes were present in all samples analyzed, while Proteobacteria and Bacteroidetes were widely distributed as well. Actinobacteria and Tenericutes were also commonly found throughout the data, but the number was relatively small. All these studies involved in the Meta‐analysis were using clone library and amplicon pyrosequencing methods. The amplification regions of 16S rRNA gene were also different. Therefore, we considered that the microbiota of *L. ridibundus* showing some similar characteristics with other avian; meanwhile, some specific features could be found for this species of avian, such as the composition of Tenericutes.

Indeed, previous studies have shown that waterfowl feces may carry human pathogens such as *Campylobacter* spp. (Broman et al., 2002; Sixl et al., 1997), *Salmonella* spp. (Baudart, Lemarchand, Brisabois, & Lebaron, 2000), pathogenic *E. coli* (Kullas, Coles, Rhyan, & Clark, 2002; Makino et al., 2000), *Microsporidia* (Slodkowicz‐Kowalska et al., 2006), and *Cryptosporidium* spp. (Zhou, Kassa, Tischler, & Xiao, 2004). The role of wild birds in transmitting pathogens indicated the importance of avian pollution in zoonosis. From our previous studies (Zhang, Yang, et al., 2016; Zhang, Zhou, et al., 2016), the most predominant bacterial pathogen in diarrhea cases in southwest China was diarrheagenic *E. coli*, followed by nontyphoidal *Salmonella*. In this study, the most frequently isolated bacteria from *L. ridibundus* were EPEC, *Salmonella* and *Aeromonas*, showing an interesting result. All the EPEC were atypical strains, lacking EAF plasmid, and the PFGE patterns showed highly heterogeneity for all the strains. However, *S. typhimurium* accounted for most of isolated *Salmonella*, and indicated highly similarly among the isolates. Makino et al. (Makino et al., 2000) found Shiga toxin (Stx)‐producing *E. coli* (STEC) strains isolated from a seagull in Japan. Kullas et al. (2002) demonstrated that Canada geese had a high prevalence of diarrheagenic *E. coli*. In the study of Palmgren et al. (2006), 83% of isolated *Salmonella* was *S. Typhimurium* from *L. ridibundus*, and indicated this wild bird might play a role in the spread of *S. Typhimurium* in Sweden. These data have paid useful attention on the risks for wild birds to public health. Although no homology of pathogenic bacteria was found between diarrhea patients and *L. ridibundus* in our study, this information was critical in order to recognize potential hazards associated with waterfowl fecal pollution. In general, this was the first study to demonstrate the microbial community structure and intestinal pathogenic bacteria from migrated *L. ridibundus* in southwest China. Firmicutes, Proteobacteria, and Tenericutes were the three most abundant phyla in the gut of *L. ridibundus*. The most isolated intestinal pathogenic bacteria from *L. ridibundus* were enteropathogenic *E. coli* and *Salmonella*. Although no homology of pathogenic bacteria was found between diarrhea patients and *L. ridibundus*, these results provided a baseline for future *L. ridibundus* microbiology study, and make an understanding of the intestinal bacterial community, structure, and diversity.

## CONFLICT OF INTEREST

The authors declare no competing financial interests.

## DATA ACCESSIBILITY STATEMENT

All data generated or analyzed during this study are included in this published article.
